# Medical Service for Convicts

**Published:** 1886-12

**Authors:** 


### MEDICAL SERVICE FOR CONVICTS.

The convicts in the Georgia penitentiary are entitled to and should have medical service furnished them by the State. Under the present arrangement the lessees employ physicians who are in no way bound to the State and who look to the lessees for their pay. They are servants of the lessee and not of the State, as they should be. The principal physician to the penitentiary, in his biennial report just made to the Governor, takes strong grounds against this. He says in his report that it is essential to the proper and humane government of the penitentiary that a State official should be at each camp—not dependent upon the lessees, or worse, the superintendent and bosses, for their position and pay, but entirely independent of them, receiving their appointment from the Governor and pay from the State.

This officer should be a physician, and, in addition to the present duties, he should be made responsible for the hygienic condition of the camp and surroundings. It should be his duty to see that the convicts are fed with proper food in quantity and quality, as directed by the principal keeper, and also see that it is properly prepared and served to the prisoners. He should be responsible for the cleanliness, ventilation and warmth in winter of the prison building and hospital, and to see that the requisite number of feet of breathing space be given each prisoner. In other words he should be made responsible for the sanitation of the camp in the broadest sense.

Without some such State official the sickness and mortality must necessarily continue, and from time to time be increased, thus bringing the blush of shame, not only to the lessees and penitentiary officials, but to every humane Georgian who is familiar with the facts.

Now give to the Penitentiary Department, in addition to the present organization, State officials, whose duty it will be to remain at the camps and see that the convicts get in full what the law provides for them, and I will here predict that the sickness and mortality will be reduced one-third to one-half within the next twelve months.

So long as the present lease system is maintained by the State of Georgia, it would appear to be the high duty of the General Assembly to perfect the law under which it is managed in all practicable methods. This recommendation, coming as it does from high authority, demands the respectful and serious consideration of that body.

